# Social and Multi-Morbidity Profiles of Older Persons With Dementia

**DOI:** 10.1093/geroni/igaf122.2582

**Published:** 2025-12-31

**Authors:** Amy Byers, Yixia Li, Ruth Morin, Daniel Jimenez, Lisa Barry

**Affiliations:** University of California, San Francisco / SFVAHCS, San Francisco, California, United States; Northern California Institute for Research and Education, San Francisco, California, United States; Hoag Memorial Hospital Presbyterian, Newport Beach, California, United States; University of Miami Miller School of Medicine, Miami, Florida, United States; University of Connecticut, Farmington, Connecticut, United States

## Abstract

Despite tremendous growth in the number of persons with dementia (PWD), little is known about the distinct social and multi-morbidity profiles that characterize PWD and whether they differ by race/ethnicity. We conducted a latent class analysis examining 653,832 PWD age ≥50 years using Medicare and U.S. Department of Veterans Affairs Veterans Health Administration data from January 1, 2012, to December 31, 2020. We triangulated multiple sources of data (e.g., geocode, health factors, VA’s Homeless Operations Management System) to identify distinct typologies of social risk and multi-morbidity. We found three distinct latent clusters defining multi-morbidity: (1) “Minimal Comorbidity” (49.6%); (2) “High Medical-Cardiac” (28.3%); (3) “Medical-High Depression/Sleep Disorder” (22.1%). We also found three clusters for social risk: (1) “Low-Moderate Social Risk” (92.1%); (2) “High Social Risk-Low Social Support/Housing Instability” (2.6%); and (3) “High Social Risk-Alcohol Abuse/Tobacco Dependence” (5.3%). These three-cluster multi-morbidity and social risk profiles differed by racial/ethnic groups. Notably, Non-Hispanic Black PWD had prominent diagnosis of posttraumatic stress disorder (over 50%) in multi-morbidity class 3, and additionally high drug abuse (over 60%) in social risk class 3. While Multiracial PWD had higher overall social risk, including high financial strain (over 60%) in social risk class 2, and Hispanic PWD had lower medical concerns in multi-morbidity class 3 but higher cardiac concerns in class 2. Establishing social and multi-morbidity profiles of PWD quantifies heterogeneity in this highly vulnerable population. These findings may help inform a more personalized, effective, and equitable approach to dementia care and provide opportunities to tailor intervention/prevention.

